# Ionomic and metabolic responses of wheat seedlings to PEG-6000-simulated drought stress under two phosphorus levels

**DOI:** 10.1371/journal.pone.0274915

**Published:** 2022-09-20

**Authors:** Li Chunyan, Zhang Xiangchi, Li Chao, Li Cheng

**Affiliations:** College of Agriculture/The Key Laboratory of Oasis Eco-agriculture, Xinjiang Production and Construction Group, Shihezi University, Shihezi, Xinjiang, P.R. China; Louisiana State University College of Agriculture, UNITED STATES

## Abstract

**Background:**

Wheat (*Triticum aestivum* L.) is a major food crop worldwide. Low soil phosphorus content and drought are the main constraints on wheat production in Xinjiang, China.

**Methods:**

In this study, the ionic and metabolic responses of one wheat variety (“Xindong20”) to drought stress simulated by using polyethylene glycol 6000 (PEG-6000) were investigated under low phosphorus (LP) and conventional phosphorus (CP) conditions by analysing wheat mineral elements and metabolites. Besides, due to xanthohumol was the metabolite with the most significant difference in expression detected in “Xindong 20”, two wheat variety “Xindong20 and Xindong 23” were selected to conduct the germination test simultaneously, to further verify the function of xanthohumol in wheat growth. Xanthohumol was mixed with PEG solution (20%) to prepare PEG solutions with different concentrations (0%, 0.1%, 0.5%, and 1%) of xanthohumol. Then wheat grains were soaked in the solutions for 20 hours, followed by a germination test. After 7 days, the indicators including shoot length, max root length, and root number were determined to identify whether the metabolite was beneficial to improve the drought tolerance of wheat.

**Results:**

The results showed that the root density and volume of wheat in LP treatment were higher than those in CP treatment. The roots underwent programmed cell death both in LP and CP treatments under PEG-6000-simulated drought stress, however, the DNA degradation in root cells in LP treatment was lower than that in CP treatment after rehydration for 3 d. Before drought stress, the malondialdehyde (MDA) content in shoot and the peroxidase (POD) activity in root in LP treatment were significantly higher than those in CP treatment, while the soluble sugar content and chlorophyll content in LP treatment were significantly lower than those in CP treatment. During drought stress, the POD activity maintained at a high level and the soluble sugar content gradually increased in LP treatment. After rehydration, the MDA content still maintained at a high level in LP treatment, the superoxide dismutase (SOD) activity increased, and the contents of soluble sugar and chlorophyll were significantly higher than those in CP treatment. The analysis of mineral elements and metabolites showed that the wheat in CP treatment was more sensitive to drought stress than that in LP treatment. Besides, the effect of drought stress was greater on shoot than on root in CP treatment, while it was opposite in LP treatment. The effect of drought stress on sugar metabolism gradually increased. Germination assays showed that 0.1% exogenous xanthohumol addition could significantly increase the shoot length of the two wheat varieties under drought stress.

**Conclusion:**

Appropriate low phosphorus supply could increase antioxidant enzyme activity in wheat, and enhance sugar metabolism to regulate osmotic balance, as well as the accumulation of various organic acids to maintain the intracellular ion homeostasis. Therefore, compared to the conventional phosphorus supply level, appropriate low phosphorus supply can significantly improve the drought tolerance of wheat. Additionally, addition of 0.1% exogenous xanthohumol, an important differential expressed metabolite in drought-stressed wheat, could effectively promote wheat shoot growth under drought stress.

## Introduction

Wheat (*Triticum aestivum* L.) is a main food crop in the world. About 70% of the wheat growing areas are distributed in arid or semi-arid areas [1], and environmental factors, such as low soil nitrogen (N) and phosphorus (P) contents and drought are the main constraints on wheat production in most wheat growing areas [2–6].

Phosphorus is one of the essential nutrients for wheat growth and development. Previous studies have reported that strategies, such as mycorrhizal symbiosis, inorganic phosphorus (P_i_) reactivation, increased synthesis of phosphatase, changing of carbon metabolism bypassing phosphorus-requiring steps, secretion of organic acids, reduced growth rate, and up-regulated expression of P_i_ transporters, have been developed by plants for P acquisition in harsh environments [7–10]. In addition, there is a significant interaction between P and drought stress. Under drought conditions, proper application of P fertilizer can significantly improve the water status and cell membrane stability of plants to maintain the normal growth and physiological process. Besides, it can also reduce drought damage and significantly improve the water use efficiency (WUE) and yield of wheat [11–13].

Metabolite profiling is rapidly becoming a key tool for gene functional annotation, enabling a comprehensive understanding of cellular responses to variations in environment conditions [14]. At present, the metabolite profiling have been performed in wheat to investigate the responses to salt [15], temperature [16], nitrogen (N) [17], and drought stresses [18]. Plant responses to drought stress involve many metabolic pathways, such as photosynthesis, sugar synthesis, tricarboxylic acid cycle, glycolysis, and hormone synthesis [19–21]. Metabolomic solutes, such as proline, betaine, fructose, and sucrose, play an important role in the drought tolerance of plants [22–24]. Besides, metabolomic components are also related to the drought tolerance of plants.

However, information regarding combined P and drought stress related metabolomic components is limited at present. Drought stress has many influences on plant metabolism, leading to, reduce water content and photosynthetic, and respiration rates, changing of enzyme activities, damage to biomacromolecules, protein degradation, cell extravasation, and growth inhibition [25]. This is closely related to ions in plants [26]. Ion balance is of great significance for maintaining the normal growth of plants, while ion imbalance may hinder plant growth. Therefore, investigating the absorption, transport, and distribution of ions in plants under drought conditions is of great significance for studying drought tolerance mechanism of plants in arid areas.

Na^+^ in soil is toxic to plants and has an antagonistic effect on the absorption of other ions (such as K^+^ and Ca^2+^), leading to nutrient deficiencies and abnormal growth of plants [27]. Previous study on the relationship between the development of primary root hairs and K^+^ flux in drought-tolerant and drought-sensitive wheat before and after drought treatment showed that the development of wheat primary root hairs was effected by net K^+^ flux. Besides, the number of primary root hairs of drought-tolerant wheat was more than that of drought-sensitive wheat for its stronger ability to absorb K^+^ [28]. Roessner and Sadiqov et al. showed that under sufficient water supply, wheat seedlings had a small amount of Ca^2+^ in their chloroplasts, However, under drought stress, the free Ca^2+^ in chloroplasts increased gradually, leading to the increased damage to the ultrastructure of chloroplasts. After rehydration, the amount of free Ca^2+^ in chloroplasts gradually decreased, and the chloroplast structure returned to normal status gradually [18, 29].

Previous studies have shown that P could obviously enhance the drought tolerance of crops and improve crop root growth and yield [30, 31], which is closely related to changes in crop physiological, biochemical, and metabolic processes and ion content. However, the ionic, physiological, biochemical, and metabolic analysis of wheat plants under combined drought and P stress has not been reported. In this study, the effects of drought stress on the physiological characteristics, metabolomics, and ion content of wheat variety “Xindong20” were investigated under two P levels, aiming to clarify the adaptive mechanism of wheat to combined drought and P stress, and screen possible markers of drought hardiness of wheat. We hypothesized that low P stress might induce the changes in the morphology, physiology, ion content, and metabolism in wheat to promote drought tolerance. This study will provide guidance for the application of P fertilizer in wheat cultivation and contribute to wheat yield increase in arid and semi-arid areas.

## Methods

### Plant materials and cultivation

The winter wheat variety “Xindong20” provided by the Agriculture College of Shihezi University was used as the experimental material. This variety has an excellent yield potential (7500–8250 kg/hm^2^) and relatively high drought tolerance and P use efficiency. Plump wheat seeds were sterilized using 1‰ mercuric chloride (HgCl_2_) solution and incubated in an incubator in the dark at 20°C after washing with distilled water. During incubation, distilled water was added every 2 d to keep moist. When seedlings had one leaf (about 7 d since sowing), sixty seedlings were transferred to one black plastic culture boxes containing the same volume of Hoagland nutrient solution, while keeping roots in the dark. There were a total of 20 boxes. The nutrient solution was changed every 2 d. Incubation under light was carried out for 18 h in each day at 25°C under photosynthetically active light (250 μmole/m^2^/s).

### Phosphorus and drought treatments

Two P levels including conventional P level (1.0 mmol/L (CP)) and low P level (0.05 mmol /L (LP)) were set in this study. Conventional P level is the P application rate adopted by our previous research that could meet the P requirements in the seedling stage of wheat, and low P level is 5% of the conventional P application level, which is insufficient for wheat growth [32]. Potassium chloride (KCl) was used to replace part of potassium dihydrogen phosphate (KH_2_PO_4_) in LP treatment. The twenty culture boxes were randomly divided into four groups. Two groups were tagged as CP treatment, with wheat seedlings cultured with Hoagland nutrient solution. The other two groups were tagged as LP treatment, with wheat seedlings cultured with low-phosphorus Hoagland nutrient solution. After 14 d of culture, polyethylene glycol 6000 (PEG-6000, osmotic potential: -0.32MPa) was mixed with Hoagland nutrient solution 15% (w/v) to simulate drought stress in CP and LP treatments. After 7 d of drought stress, Hoagland nutrient solution without PEG-6000 was used to rehydrate for 3 d. Then, one part of seedlings in each group was used to determine the physiological and biochemical indexes, and the other part was used to determine the metabolites and ion content. Seedlings were sampled at 0 d (DSD 0), 3 d (DSD 3), 5 d (DSD 5), and 7 d (DSD 7) under drought stress and 3 d after rehydration (RD 3).

### Biomass measurement

The shoot and root of wheat in different treatments were sampled at DSD 0, DSD 3, DSD 5, DSD 7, and RD 3. Part of them was dried at 70°C to constant weight and weighed. The other part was directly weighed to determine the fresh weight. At least five biological replicates were used for biomass measurement.

### Root morphology

Clean root samples were spread out completely while avoiding overlap and scanned by using the root scanning system (Wanshen LS-A, Phantom 9850XL PLUS, China) according to the manufacturer’s instructions. WinRHIZO PRO2009 software (Regent Instrumment Inc., Canada) was used to analyze the average root diameter and total root volume.

### DNA extraction and fragment analysis

DNA was extracted from frozen root tip by using EasyPure Plant Genomic DNA kits (Transgen, Code#9192EE111-01, China) according to the manufacturer’s instructions. The quality and concentration of genomic DNA were measured using a nucleic acid protein analyzer (NanoDrop, ND-1000, USA). The sample amount was the same in each treatment in each period. Ethidium bromide staining and 1.5% agarose gel electrophoresis were performed to visualize the DNA fragments.

### Determination of physiological and biochemical indexes

The chlorophyll content in shoot was determined by ethanol extraction colorimetry [33], and the contents of soluble sugar (SS), and malondialdehyde (MDA) in shoot and root were determined by anthrone colorimetry [33] and thiobarbituric acid (TBA) colorimetry [32], respectively. The superoxide dismutase (SOD) activity and peroxidase (POD) activity in shoot and root were determined according to Wang’s methods [34].

### Sample preparation and untargeted metabolomic analysis

Metabolites in root and shoot were analyzed by using the liquid chromotography with mass spectrometry (LC-MS), with the assistance of BGI Biotechnology Corporation, China. Briefly, samples (25 mg) stored at -80°C were transferred in EP tubes, and then 800 μL of chilled methanol/water (1:1) buffer solution and two small steel balls were added to each EP tube. Then, the samples were ground by using Tissuelyser (Qiagen, Germany) at 55 Hz for 4 min. After taking out the steel balls, the tubes was placed at -20°C for precipitation, and then centrifuged at 30,000×g for 20 min at 4°C. The supernatant (650 μL) in each tube was transferred to a new tube. And 20 μL of the supernatant of each sample was mixed to prepare QC samples, which were subjected to LC-MS analysis. Five biological replicates were set in this analysis.

The samples prepared in above step were injected onto the LC column in splitless mode using the LC-MS 2777C UPLC system (Waters, UK). To reduce system error, the samples were randomized. Data acquirement was completed in one day. The LC column used was ACQUITY UPLC CSH C18 (100 mm × 2.1 mm, 1.7 μm, Waters, UK). The main settings were as follows: mobile phase A: 0.1% acetonitrile/water (60: 40), and mobile phase B: isopropyl alcohol/ acetonitrile (90: 10). The injection volume was 10 μL. The flow rate was 0.4 mL/min. The column temperature was 40°C. The gradient elution procedure was as follows: 0–2 min, 60% A, 40% B; 2–2.1 min, 57% A, 43% B; 2.1–6 min, 50% A, 50% B; 6–6.1 min, 46% A, 54% B; 6.1–8 min, 30% A, 70% B; 8–8.1 min, 1% A, 99% B; 8.1–10 min, 60% A, 40% B. Electrospray ionization (ESI+/ESI-) mode was used for mass spectrometry, and the scanning mode adopted positive and negative ion modes. The capillary voltage was 0.25 Kv (+) / 2 Kv (-).

### Ion content analysis

Wheat seedlings with uniform growth were randomly collected from CP and LP treatments (five biological replicates). Wheat shoot and root were subjected to ion content analysis by using Agilent ICP-OES 710 (Agilent, USA). The standard solution (1000 μg/mL) was diluted with 2% nitric acid to prepare the standard curve. Briefly, the oven-dried sample of 0.1 g was put in a polytetrafluoroethylene beaker, and then 10 mL of nitric acid was added. The mixture was heated on a hot plate for 12 h, and then digested at 150°C until 2 ~ 3 mL of liquid remained. The remaining liquid was cooled, transferred into 20 mL volumetric flask, and diluted with 2% nitric acid to constant volume. Meanwhile, a blank test without the addition of sample was done.

### Germination test

In the analysis of metabolites, it was found that in “Xindong 20”, the content of xanthohumol in wheat shoot in LP treatment was 5.019 times higher than that in CP treatment. Therefore, we conducted germination test to determine the function of xanthohumol in regulating wheat growth through exogenous addition of different concentrations of xanthohumol. Besides, due to xanthohumol is the metabolite with the most significant difference in expression detected in “Xindong 20”, we selected the other wheat variety “Xindong 23” to conduct the germination test simultaneously, to further verify the function of xanthohumol in wheat growth.

Wheat varieties “Xindong20” and “Xindong23” ("Xindong20" has higher drought tolerance and P use efficiency than "Xindong23") were selected for germination test. The experiment had a control group (distilled water) and a 20% PEG-6000 treated group (PEG group). For the PEG group, wheat seeds were respectively soaked in PEG solutions (20%, w/v) containing different concentrations of xanthohumol (0, 0.1%, 0.5%, and 1%) for 20 h, followed by a germination test in germination box at 25°C on a 16 h light / 8 h dark cycle During this period, the same volume of PEG solution (20%) was added to keep moist. After 7 days, the shoot length, maximum root length, and root number were determined. Three repetitions were set for each group.

### Statistical analysis and image processing

The data were analyzed using Microsoft Excel (version 16.0, Microsoft, USA) and SPSS software (version 13.0, IBM, New York, USA). Each measurement was repeated at least three times. T-tests were performed for pairwise comparisons by using SPSS software (version 13.0, IBM, New York, USA).

In the statistical analysis, the p-value produced by rank sum test was further corrected by false discovery rate (FDR) to obtain the q-value. The thresholds for screening differential expressed metabolites (DEMs) were the fold change greater than 1.2 or less than 0.8 and q-value less than 0.05. Peak extraction, including peak alignment, peak extraction, normalization, deconvolution, and compound identification, was realized by using Progenesis QI software (Version 2.0, Waters, UK). Additionally, to avoid false-positive results in the screening for DEMs, the significance criterion of FDR was used and an FDR limit of 0.05 was selected. To perform a comparison between different treatments, the detected metabolites were subjected to a principal components analysis (PCA) and Pareto-scaled, and the key metabolites were identified by partial least-squares discriminant analysis (PLS-DA).

## Results

### Morphological and physiological characteristics of wheat root

**Fig 1:**
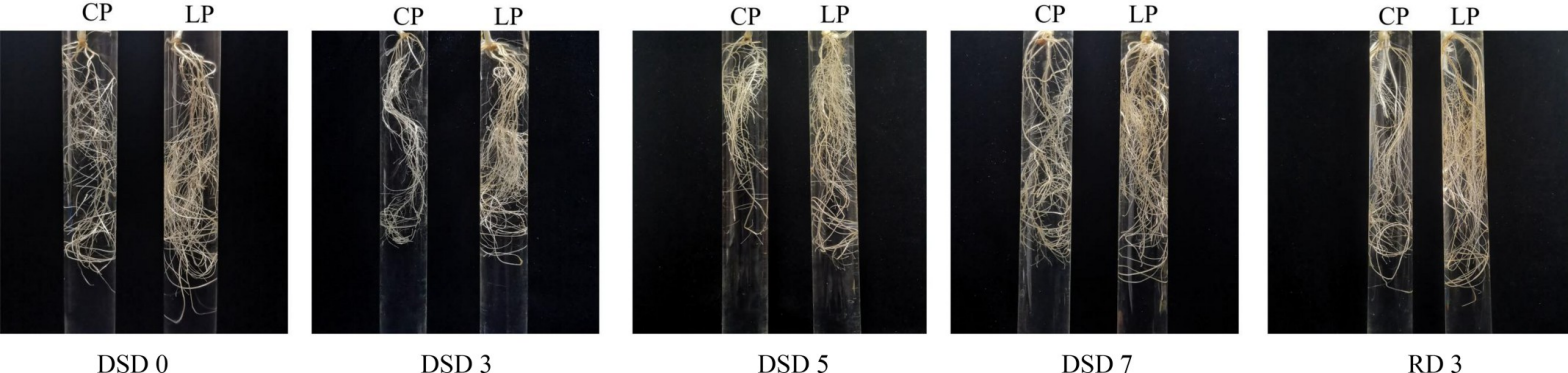
Morphology of wheat roots in CP and LP treatments under drought stress. Note: CP: conventional phosphorus; LP: low phosphorus.

Phosphorus application had a significant effect on wheat root growth. At DSD 0, the root in LP treatment was thicker than that in CP treatment, and the root color was deeper than that in CP treatment. Besides, root tip enlargement was observed at the tips of each root and bifurcate root in the two treatments, but it was more obvious in CP treatment than in LP treatment (Fig 1).

**Fig 2:**
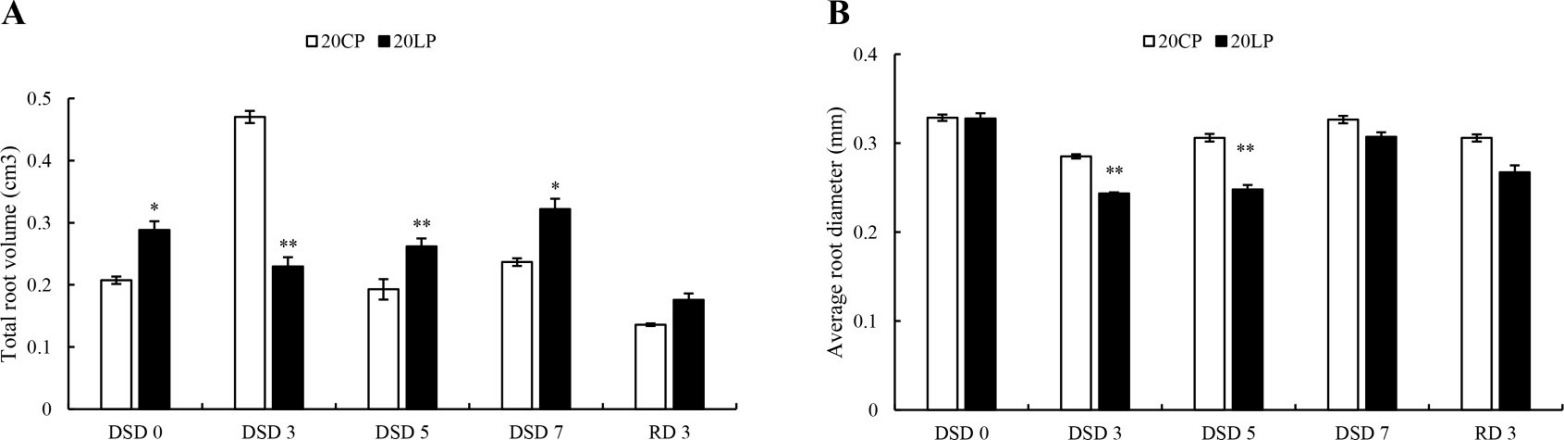
The total volume and average diameter of wheat roots. Note: CP: conventional phosphorus; LP: low phosphorus; Values are means±standard deviation of three repetitions; * and ** indicate significantly different at *p* < 0.05 and *p* < 0.01, respectively. 20CP: Xindong20 under conventional phosphorus level; 20LP: Xindong20 under low phosphorus level.

At DSD 0, 5, and 7, the total root volume in LP treatment was significantly higher than that in CP treatment (Fig 2), but there was no significant difference in the average root diameter at DSD 0, DSD 7, and RD 3.

**Fig 3:**
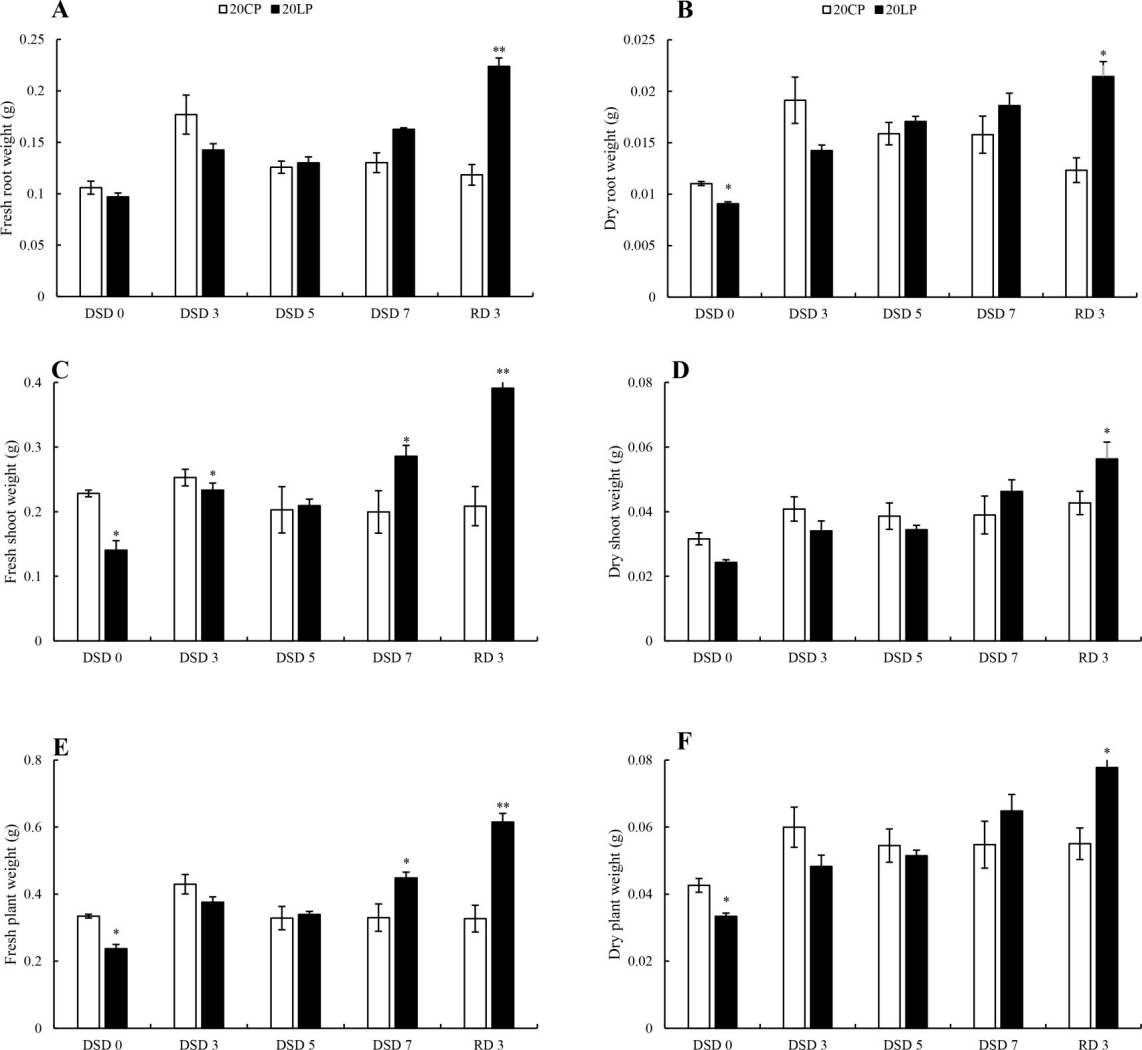
The fresh and dry weight of wheat plants. Note: CP: conventional phosphorus; LP: low phosphorus; Values are means±standard deviation of three repetitions; * and ** indicate significantly different at *p* < 0.05 and *p* < 0.01, respectively. 20CP: Xindong20 under conventional phosphorus level; 20LP: Xindong20 under low phosphorus level.

From DSD 0 to DSD 3, the wheat dry weight and fresh weight in LP and CP treatments showed a gradual increasing trend, but the dry weight and fresh weight of root, shoot, and whole plant in LP treatment were lower than those in CP treatment. From DSD 5 to RD 3, the dry weight and fresh weight in LP treatment increased significantly compared with those in CP treatment. However, the fresh weight and dry weight of root in CP treatment showed a downward trend. Especially at RD 3, the dry weight and fresh weight in LP treatment were significantly higher than those in CP treatment (Fig 3).

### DNA laddering

**Fig 4:**
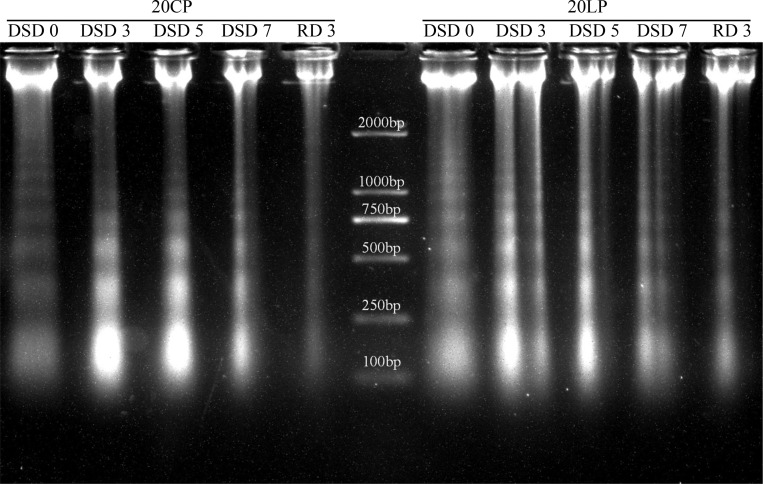
DNA fragmentation analysis. Note: 20CP: Xindong20 under conventional phosphorus level; 20LP: Xindong20 under low phosphorus level. DSD 0, DSD 3, DSD 5 and DSD 7 refer to days 0, 3, 5 and 7 under drought stress, respectively. RD 3 refers to day 3 after rehydration.

To investigate the effect of drought stress on programmed cell death in root in CP and LP treatments, DNA extracted from root tip was used to perform gel electrophoresis. The results showed that DNA ladder was gradually obvious from DSD 0 to DSD 5 (Fig 4), and the DNA ladder intensity in CP treatment was stronger than that in LP treatment. It was worth noting that the ladder intensity at DSD 7 was not the strongest in LP and CP treatments. At RD 3, the DNA ladder of low molecular weight DNA was the weakest in the two treatments, while that of high molecular weight DNA in LP treatment was significantly stronger than that in CP treatment.

### Physiological and biochemical characteristics of wheat

**Fig 5:**
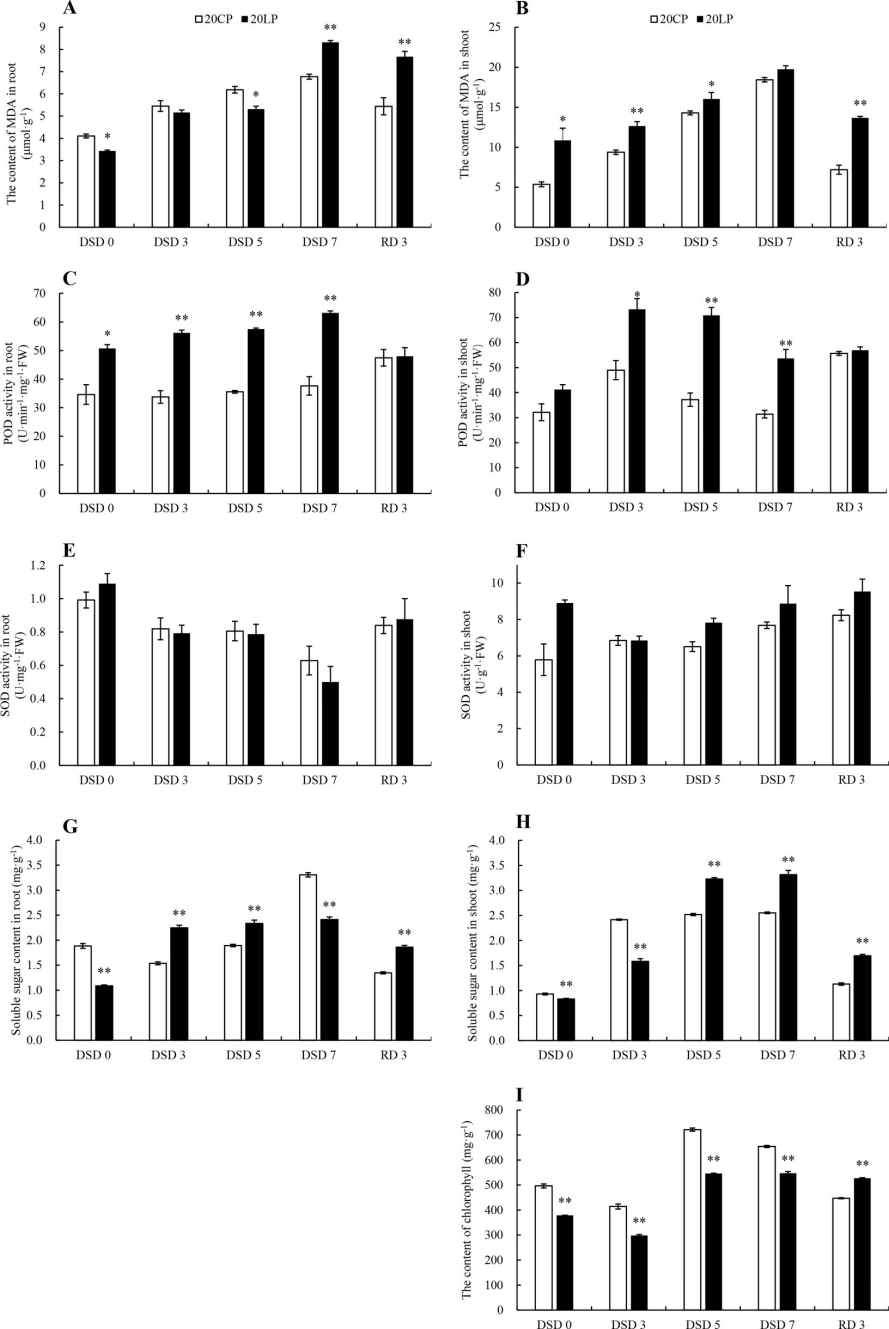
Physiological and biochemical indexes of wheat plants. Note: CP: conventional phosphorus; LP: low phosphorus; Values are means±standard deviation of three repetitions; * and ** indicate significantly different at *p* < 0.05 and *p* < 0.01, respectively. 20CP: Xindong20 under conventional phosphorus level; 20LP: Xindong20 under low phosphorus level. MDA: malondialdehyde; SOD: superoxide dismutase; POD: peroxidase.

The MDA content in root increased gradually in CP and LP treatments from DSD 0 to DSD 7, and the peak value in LP treatment was significantly higher than that in CP treatment. From DSD 7 to RD 3, the MDA content in root in LP and CP treatments decreased by 7.8% and 19.7%, respectively (Fig 5A). The change of MDA content in shoot was similar to that in root, but the MDA content in LP treatment was significantly higher than that in CP treatment from DSD 0 to DSD 5 (Fig 5B).

From DSD 0 to DSD 7, the POD activity in root gradually increased in LP treatment, which was also significantly higher than that in CP treatment (Fig 5C). However, there was no significant difference in CP treatment during this period. From DSD7 to RD 3, the POD activity in LP treatment decreased suddenly, while that in CP treatment increased slightly; the difference between them was not significant. The POD activity in shoot increased first and then decreased from DSD 0 to DSD 7, and it was significantly higher in LP treatment than in CP treatment from DSD 3 to DSD 7 (Fig 5D).

The SOD activity in root decreased gradually under drought stress and then increased after rehydration (Fig 5E). There was no significant difference between LP and CP treatments. The SOD activity in shoot showed a small upward trend (Fig 5F).

From DSD 0 to DSD 7, the content of soluble sugar in root increased gradually in CP treatment, with peak value appearing at DSD 7 (Fig 5G), while that in LP treatment remained relatively stable from DSD 3 to DSD 7. From DSD7 to RD 3, the content of soluble sugar in root in CP treatment decreased sharply, but it was still significantly higher than that in LP treatment. The change of soluble sugar content in shoot was similar to that in root, but the peak value in LP treatment was significantly higher than that in CP treatment (Fig 5H).

The chlorophyll content in shoot decreased first and then increased from DSD 0 to DSD 7, and it was significantly higher in CP treatment than in LP treatment. At RD 3, the chlorophyll content in shoot in LP treatment was significantly higher than that in CP treatment (Fig 5I).

### Metabolic profiling of wheat under combined phosphorus and drought stress

To understand in more detail the difference in wheat responses to drought stress under two phosphorus levels, metabolites in wheat shoot and root were detected by LC-MS/MS (S1 File). The ions with RSD < 30% in QC samples were used for further analysis. The results showed that under positive ion mode, the expression of 64 metabolites in root were up-regulated and that of 38 metabolites were down-regulated (102 DEMs totally) at DSD 3, involving 10 differentially expressed pathways. In addition, no DEM was detected in shoot at DSD 3 and DSD 7 and in root at DSD 7. Under negative ion mode, the expression of 9 metabolites were up-regulated and that of 2 were down-regulated in shoot (11 DEMs totally) at DSD 3, involving 2 differentially expressed pathways. At DSD 7, the expression of 8 metabolites were up-regulated and that of 38 were down-regulated in root, involving 19 differentially expressed pathways (46 DEMs totally). In addition, no DEMs were detected in root at DSD 3 and in shoot at DSD 7. The number of DEMs in root was higher than that in shoot, indicating that the root was more sensitive to the stress environment. The difference between root and shoot was greater than that between different sampling dates.

In this study, the metabolites in root and shoot of wheat in CP and LP treatments sampled at DSD 3 and DSD 7 were studied by metabolomics method. It was found that there were significant differences in metabolites in wheat root and shoot in LP and CP treatments(S2 File). In CP treatment, 67 differential metabolic pathways involving 598 DEMs were detected in shoot at DSD 3 compared to those at DSD 7 (S1 Table), while no DEMs were detected in LP treatment. The DEMs in CP treatment were involved in 8 metabolic pathways, and most of them were up-regulated in root (S2 Table). There were 120 DEMs in LP treatment, involving 39 metabolic pathways, and most of them were up-regulated in root (S3 Table).

At DSD 3, compared with CP treatment, only 2 differential metabolic pathways were detected in shoot in LP treatment. The first pathway was flavonoid biosynthesis, and the DEM involved was xanthohumol, with a significant increase of 5.019 fold. The other differential metabolic pathway was phenylpropanoid biosynthesis, and the DEM involved was 1-O-Sinapoyl-β-D-glucose, with a significant increase of 4.469 fold.

LC-MS-based metabolomic analysis showed that there were 10 differential metabolic pathways involving 27 DEMs in wheat root in LP and CP treatments at DSD 3. Among them, pathways including strictosidine aglycone, horhammericine, dialdehyde, strictosidine aglycone, and 11-deoxycorticosterone had the highest fold change, reaching 1.85 fold. The other metabolites had fold change less than 1 (Table 1).

**Table 1:** Differentially expressed metabolites in roots of Xindong 20 at day 3 under simulated drought stress in LP and CP treatments*.

Metabolic pathway	Metabolite	Fold change (LP/CP)	Metabolic pathway	Metabolite	Fold change (LP/CP)
Indole alkaloid biosynthesis	Strictosidine aglycone	1.85	Sesquiterpenoid and triterpenoid biosynthesis	Solavetivone	0.42
	Horhammericine	1.85		Pentalen-13-al	0.4
	Dialdehyde	1.85		Albaflavenone	0.42
Phenylpropanoid biosynthesis	1-O-Sinapoyl-β-D-glucose	0.58	Metabolic pathways	5’-Methylthioadenosine	0.23
Plant hormone signal transduction	Abscisate	0.51		Strictosidine aglycone	1.85
Carotenoid biosynthesis	Abscisic acid	0.51		11-Deoxycorticosterone	1.85
Cysteine and methionine metabolism	5’-Methylthioadenosine	0.23		Abscisate	0.51
Zeatin biosynthesis	O-β-D-Glucopyranosyl-cis-zeatin	0.58		17α-Hydroxypregnenolone	0.52
	trans-Zeatin-7-β-D-glucoside	0.58	Isoquinoline alkaloid biosynthesis	Magnoflorine	0.59
	O-β-D-Glucosylzeatin	0.58			
	5’-Methylthioadenosine	0.23			

*Note: The pathway of biosynthesis of secondary metabolites involved 7 differential expressed metabolites was not shown.

The KEGG enrichment analysis showed that there were 80 DEMs, involving 19 metabolic pathways in wheat root in LP and CP treatments at DSD 7. The DEMs arranged in descending order of quantity were: metabolic pathways, biosynthesis of secondary metabolites, fructose and mannose metabolism, amino sugar and nucleotide sugar metabolism, galactose metabolism, carbon metabolism, inositol phosphate metabolism, limonene and pinene degradation, starch and sucrose metabolism, glycolysis/ gluconeogenesis, pentose phosphate pathway, phosphatidylinositol signaling system, ascorbate and aldarate metabolism, biosynthesis of amino acids, carbon fixation in photosynthetic organisms, phenylalanine, tyrosine and tryptophan biosynthesis, pentose and glucuronate interconversions, ABC transporters, cutin, suberine and wax biosynthesis (Table 2). All of them had fold change (LP/CP) less than 1.

**Table 2:** Differentially expressed metabolites in roots of Xindong 20 at day 7 under drought stress in LP and CP treatments*.

Metabolic pathway	Metabolite	Fold change (LP/CP)	Metabolic pathway	Metabolite	Fold change (LP/CP)
Phosphatidylinositol signaling system	Inositol 1-P	0.058	Carbon metabolism	D-arabino-Hex-3-ulose 6-P	0.058
	myo-Inositol 4-P			β-D-Fructose 6-P	
	1D-myo-Inositol 3-P			α-D-Glucose 6-P	
Inositol phosphate metabolism	D-Glucose 6-P	0.058		D-Fructose 6-P	
	Inositol 1-P			β-D-Glucose 6-P	
	myo-Inositol 4-P		Pentose and glucuronate interconversions	D-Glucose 1-P	0.058
	1D-myo-Inositol 3-P		Starch and sucrose metabolism	D-Glucose 6-P	0.058
Ascorbate and aldarate metabolism	L-Gulose 1-P	0.058		D-Glucose 1-P	
	β-L-Galactose 1-P			D-Fructose 6-P	
Amino sugar and nucleotide sugar metabolism	α-D-Galactose 1-P	0.058		β-D-Glucose 1-P	
	D-Mannose 6-P		ABC transporters	Inositol 1-P	0.058
	β-D-Fructose 6-P		Glycolysis / Gluconeogenesis	β-D-Fructose 6-P	0.058
	α-D-Glucose 6-P			α-D-Glucose 6-P	
	D-Glucose 1-P			D-Glucose 1-P	
	D-Mannose 1-P			β-D-Glucose 6-P	
Carbon fixation in photosynthetic organisms	D-Fructose 6-P	0.058	Biosynthesis of amino acids	D-arabino-Hex-3-ulose 6-P	0.058
Phenylalanine, tyrosine and tryptophan biosynthesis	D-Fructose 1-P	0.058		β-D-Fructose 6-P	
Limonene and pinene degradation	(3S)-6-Hydroxy-3-isopropenyl-heptanoate	0.12	Cutin, suberine and wax biosynthesis	16-Oxo-palmitate	0.17
	(5R)-6-Hydroxy-5-isopropenyl-2-methylhexanoate		Fructose and mannose metabolism	D-Mannose 6-P	0.058
	(3R)-6-Hydroxy-3-isopropenyl-heptanoate			β-D-Fructose 6-P	
	(5S)-6-Hydroxy-5-isopropenyl-2-methylhexanoate			D-Allose 6-P	
Galactose metabolism	α-D-Galactose 1-P	0.058		β-D-Fructose 2-P	
	D-Tagatose 6-P			D-Fructose 1-P	
	α-D-Glucose 6-P			Sorbose 1-P	
	D-Glucose 1-P			D-Mannose 1-P	
	D-Galactose 6-P		Pentose phosphate pathway	D-arabino-Hex-3-ulose 6-P	0.058
				β-D-Fructose 6-P	
				α-D-Glucose 6-P	
				β-D-Glucose 6-P	

*Note: The pathways of biosynthesis of secondary metabolites involved 8 differential expressed metabolites and the metabolic pathway involved 17 differential expressed metabolites were not shown.

### Changes in ion content in wheat root and shoot

**Fig 6:**
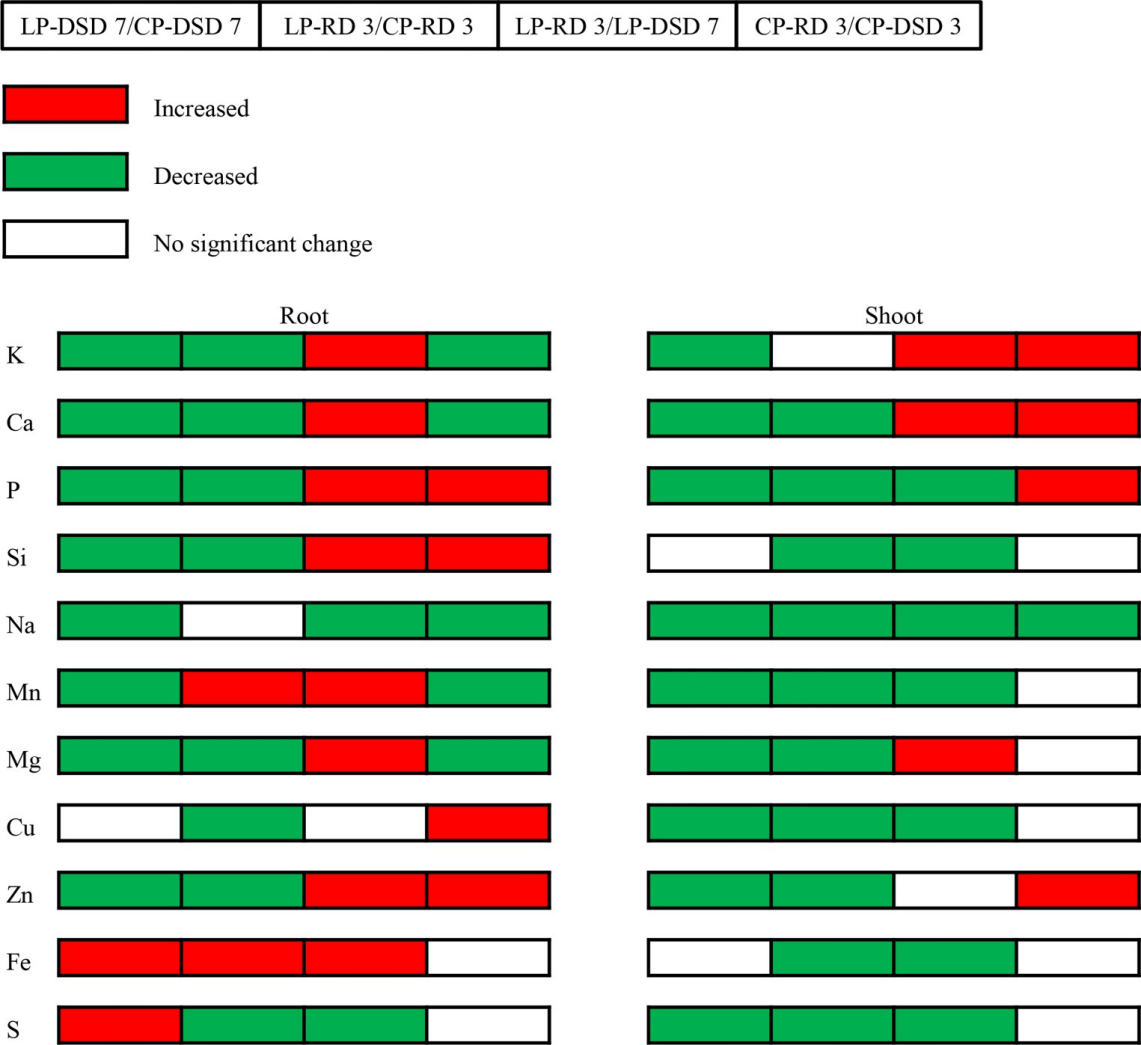
Changes of mineral elements in wheat roots and shoots. Note: Red boxes refer to significantly enhanced elements, whereas green boxes refer to significantly reduced elements (*p* < 0.05). CP: conventional phosphorus; LP: low phosphorus; DSD 3, DSD 7 and RD 3 refer to days 3 and 7 under drought stress and day 3 after rehydration, respectively.

To explore the response of wheat to drought stress at two phosphorus levels, the changes of 11 ions in root and shoot were measured by mass spectrometry at DSD 7 and RD 3 (Fig 6). The results showed that the contents of potassium (K), calcium (Ca), P, silicon (Si), sodium (Na), manganese (Mn), magnesium (Mg), and zinc (Zn) in root in LP treatment significantly decreased compared with those in CP treatment at DSD 7, especially the contents of P (85.8%), Mn (69.3%), Zn (62.5%), and Mg (62.1%). However, the contents of iron (Fe) and sulfur (S) significantly increased by 79.6% and 37.9%, respectively. The change in copper (Cu) content was not significant. At RD 3, the contents of K, Ca, P, Si, Mg, Cu, Zn, and S in LP treatment significantly decreased compared with those in CP treatment, especially the contents of P (68.6%), Zn (51%), and Cu (47.5%). However, the contents of Mn and Fe significantly increased by 53.8% and 208.2%, respectively compared with those in CP treatment. There was no significant difference in Na content. At RD 3, the contents of K, P, Si, Mn, Mg, Zn, Ca, and Fe in LP treatment significantly increased by 42.5%, 155.3%, 102.9%, 120.4%, 75.3%, 81.1%, 21.0%, and 60.14%, respectively, while the contents of Na and S significantly decreased by 38% and 46.1%, respectively, compared with those at DSD 7. There was no significant difference in Cu content. In CP treatment, the contents of P, Si, Cu, and Zn significantly increased by 15.6%, 109.1%, 82.1%, and 38.3%, respectively at RD3 while the contents of K, Na, Mn, Ca, and Mg significantly decreased by 24.1%, 67.4%, 56.1%, 19.27%, and 10.5%, respectively compared with those at DSD 7. There was no significance in the contents of Fe and S.

### Effects of xanthohumol on related indicators of wheat seedling under drought stress

**Fig 7:**
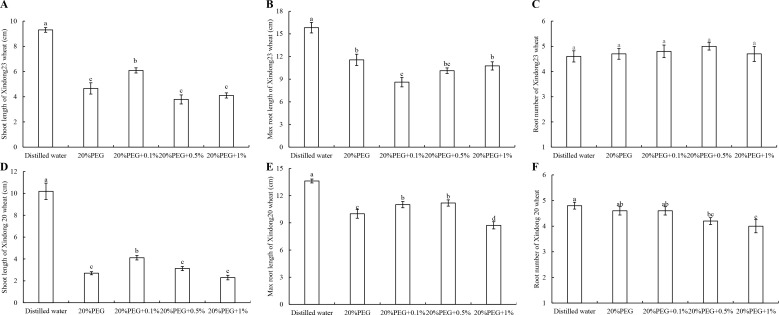
Effects of exogenous xanthohumol addition on germination rate of wheat seeds under PEG-6000-simulated drought stress. Note: A, B, and C represent the shoot length, max root length, and root number of Xingdong23 wheat, respectively. D, E, and F represent the shoot length, max root length and root number of Xingdong20 wheat, respectively. Different lowercase letters represent significantly different at *p* < 0.05.

As mentioned above, the content of xanthohumol in shoot in LP treatment was 5.019 times higher than that in CP treatment. To investigate whether exogenous xanthohumol addition can improve wheat drought tolerance, wheat varieties “Xindong20” and “Xindong23” were selected for germination test. The xanthohumol treatment had no significant effect on the germination rate of wheat seeds (“Xindong20” and “Xindong23”) under drought stress. However, 0.1% xanthohumol addition could significantly increase the shoot length of “Xindong 23” and “Xindong20” by 30.7% and 52.4%, respectively under drought stress, compared with 0% xanthohumol-PEG group (Fig 7A and 7D). Compared with the PEG group without xanthohumol addition, the maximum root length in 0.1% xanthohumol-PEG group was significantly reduced by 25.5% in “Xindong 23” (Fig 7B) and increased by 10.1% in “Xindong20” (Fig 7E). Besides, xanthohumol addition had no significant effect on the root number of “Xindong23” (Fig 7C), but the root number of “Xindong20” reduced with the increase of xanthohumol concentration (Fig 7F). The root number of “Xindong20” in 1% xanthohumol-PEG group reduced by 13.04% compared with that in the PEG group without xanthohumol addition.

## Discussion

### Effects of simulated drought stress on wheat root morphology and physiological indexes under two phosphorus levels

In this study, from DSD 5 to RD 3, low P supply promoted plant dry matter accumulation and root development. This indicates that appropriate low P supply could improve wheat growth under drought stress. This is consistent with the results of previous studies [35, 36]. The change of root architecture in LP treatment can be attributed to changes of auxin, cytokinin, and ethylene [37–42]. Besides, this study found that some metabolic pathways and metabolites related to plant hormone synthesis and P response changed significantly, such as plant hormone signal transduction, alkaloid synthesis, and abscisic acid. This may be due to that with the growth of lateral roots, the responses of cell wall synthesis and growth-related genes to P accelerate. For example, the gene chip detection in our previous study found that the expression of the gene encoding cell wall invertase in wheat root treated with LP and 7 days of drought stress increased significantly [43].

In this study, it was found that root tip swelling was detected after 7 days of drought stress under the two phosphorus levels, and it was more obvious in CP treatment than LP treatment. This may be related to the endopolyploidy in root cells [44]. Previous study has found that zeatin can affect the cell cycle by regulating cyclin CycD3 [44], and phosphate stress can also affect the cell cycle, resulting in endopolyploidy in plants [45]. In this study, after 3 days of drought stress, the ratio of zeatin biosynthesis related DEMs in LP treatment to that in CP treatment was less than 1. This indicates that LP may induce endopolyploidy in root cells. In addition, sucrose is the main carbon source in plants and plays an important role in regulating the cell cycle [46]. In this study, sucrose metabolic pathway was inhibited in LP treatment. This may reduce sugar yield, inhibit cell division, and reduce endopolyploidy in root cells. The reduction of endopolyploidy is helpful to reduce the input of carbon source and other nutrients. In addition, under the limited P conditions, the reduction of endopolyploidy in lateral roots helps to reduce P consumption in the synthesis of phosphorylated metabolites such as genomic DNA and RNA, which ultimately helps plants better adapt to the environment [45]. In this study, the detection of root tip DNA ladder also showed that although the roots underwent programmed cell death under both P levels from DSD 0 to DSD 7, the degradation level of DNA in root cells was significantly weaker in LP treatment than in CP treatment at RD 3. This indicates that low P is more beneficial to the rapid recovery of drought-stressed wheat after rehydration than CP. This is also confirmed by our results of root morphology and dry matter accumulation.

In this study, the content of MDA in shoot was higher in LP treatment than in CP treatment (Fig 5B). This indicates that the combined low phosphorus and drought stresses could cause more serious damage to plants. However, at the same time, it could also make plant shoot accumulate more antioxidant enzymes such as POD and SOD and soluble sugar (Fig 5D, 5F and 5H), thus reducing oxidative stress, maintaining cell osmotic balance, and improving the utilization efficiency of nutrients and water (Fig 5B). Studies have shown that exogenous silicon application can alleviate the oxidative stress of many plant species [47–51]. Tripathi et al. found that exogenous silicon application can enhance the tolerance to ultraviolet stress and the activity of antioxidant enzymes, such as SOD, ascorbate peroxidase (APX), and catalase (CAT) in wheat [52]. Although no exogenous silicon was applied in this study, the content of silicon in roots in CP and LP treatments at RD 3 significantly increased by 70.4% and 95.8%, respectively compared with those at DSD 7. It indicates that stress environment could induce the changes of the absorption and displacement of silicon in plants to improve the stress tolerance of plants and maintain root activity. This may be one of the reasons why the recovery of wheat in LP treatment is faster than that in CP treatment.

### Effects of drought stress on ion content of wheat plants under different phosphorus levels

Under drought stress, plant water metabolism is seriously affected, and ion metabolism closely related to water metabolism is also affected [26]. In this study, the contents of most ions in wheat root decreased significantly after 7 days of drought stress and 3 days of rehydration in LP treatment (Fig 6). Only the contents of Fe (DSD 7), S (DSD 7), Mn (RD 3), and Fe (RD 3) increased significantly. In addition, it was observed that the roots of wheat seedlings turned yellow after 7 days of drought. This indicates root damage has occurred, which reduces the absorption capacity of wheat roots. Watt et al. found that under drought stress, the accumulation of hydrophobic substances on the cell wall of plant root epidermal cells may affect the absorption of mineral elements in root apoplasts and transmembrane transport, resulting in the decrease of most ion contents [53]. Besides, the increases of Mn and Fe contents in LP treatment may be due to the increased water loss transpiration rate, root/shoot ratio, and absorption capacity of wheat seedlings caused by drought stress.

In this study, the content of most ions in shoot showed a downward trend in CP and LP treatments. Although most of the ions in root also decreased, the number of ions decreased in shoot was greater than that in root (Fig 6). This indicates that under drought stress, P supply has a greater impact on wheat shoot than on wheat root. Besides, it was observed that the leaves of wheat seedlings turned yellow and even withered seriously under drought dress. The contents of P, Si, Na Mn, Cu, Fe, and S significantly decreased from DSD 7 to RD 3 in LP treatment. However, the contents of K, Ca, P, and Zn increased and not significant difference was found in the contents of six ions (Si, Mn, Mg Cu, Fe, and S) in CP treatment (Fig 6). This indicates that under the same conditions, drought can strengthen the effect of low phosphorus stress [42]. In CP treatment, the sufficient P supply significantly improves the drought resistance of wheat. It can be seen that the response of wheat shoot to stress environment is different from that of root.

As mentioned above, LP could improve the adaptability to drought stress and maintain ion balance of wheat. When wheat is under drought stress, the ion balance is destroyed, but the sensitivity is reduced. After rehydration, the contents of the ions related to stress tolerance in root increased significantly, such as K, P, Si, Mn, Mg, Zn, Ca, and Fe (Fig 6). Similarly, the contents of K and Ca in shoot also increased significantly after rehydration. K^+^ could improve the activities of most enzymes in plant metabolism, and Ca^2+^ can improve the osmotic pressure in plants [27, 28]. The increased uptake and accumulation of K^+^ and Ca^2+^ by wheat can reduce the adverse effects of drought stress on plant growth and enhance the tolerance of plants to drought stress [54]. Under the two P levels, the Si content increased significantly from DSD 7 to RD 3. This may promote root growth, and increase root vitality and the absorption of water and nutrients by plants [55]. It should be noted that except for RD 3, the content of Na decreased significantly in CP and LP treatments, but the decrease in LP treatment was lower than that in CP treatment from DSD 7 to RD 3 (Fig 6). It may be due to that the increase of root external protons after rehydration enhances the Na^+^ reverse transport in root plasma membrane and the excretion of Na^+^ [56]. The lower variation of Na^+^ content in LP treatment than in the control group may be related to the reduced sensitivity of wheat to drought caused by LP.

### Effects of drought stress on wheat metabolome under two phosphorus levels

There were significant differences in metabolites in drought-stressed wheat root and shoot between LP and CP treatment in this study. Drought stress had a greater effect on wheat in CP treatment than on wheat in LP treatment. Besides, the effect of drought stress on shoot was greater than that on root in CP treatment, while the effect on root was greater than that on shoot in LP treatment. Taking the common ABC transporter pathway in root at DSD 3 and 7 as an example, the ABC transporter pathway in CP treatment mainly involved sugars, especially maltose and maltodextrin, while the main sugars involved in LP treatment were xylose, arabinose, ribose, and arginine. In plants, sugars are produced through photosynthesis, polysaccharide degradation, and gluconeogenesis [44]. The difference in the contents of sugars and other carbohydrates between drought-stressed plants and sufficiently irrigated plants are considered to be metabolic signals in arid environment of plants [18]. In wheat leaves, sugars play a main role in regulating infiltration. Under short-term drought conditions, starch synthesis is more easily inhibited than sucrose synthesis [6]. In this study, the metabolic pathways of starch and sucrose in wheat root changed significantly from DSD 3 to DSD 7 in CP treatment, but no significant change was detected in LP treatment. At the same time, it was found that the number of DEMs related to sugar metabolic pathway was more in CP treatment than in LP treatment. This indicates that the wheat under CP was more sensitive to drought stress. In addition, at DSD 3, compared to CP treatment, the differential metabolic pathways in LP treatment did not involve glucose metabolism, while at DSD 7, the DEMs in pathways related to sugar metabolism such as galactose metabolism, pentose and gluconate interconversions, starch and sucrose metabolism, glycolysis/gluconeogenesis, and fructose and mannose metabolism decreased significantly. This indicates that with the prolongation of drought stress, the effect of drought stress on sugar metabolism is greater. The stronger adaptability of wheat to drought stress in LP treatment than in CP treatment may be due to that the great enhancement of sugar metabolism is beneficial to maintaining osmotic balance.

Previous studies have shown that there are a lot of starch in plant root cap cells, and the drought tolerance of crops could be judged according to the amount of starch residue in seedling root cap cells after stress [57]. According to the results of our previous gene chip study, at DSD 7, the expression of beta amylase gene of the starch degrading enzyme was significantly up-regulated in LP treatment, while the expression of starch branching enzyme III gene and glycogen (star) synthase gene of the starch synthesis related enzymes were significantly down-regulated [42], compared with those in CP treatment. This indicates the acceleration of the degradation of starch and the decreased rate of starch synthesis in root in LP treatment. The result of this study that the ratio of glucose-1-phosphate (the precursor of starch synthesis) in CP treatment to that in LP treatment is less than 1 further confirmed it (Table 2). Therefore, in this study, the degradation of starch in plant root cap cells may occur and the root system may be dehydrated. Besides, the dehydration may be more serious in LP treatment than in CP treatment. The results of the root DNA fragment and root water content also confirmed it. Therefore, it is speculated that the degradation of polysaccharides may be enhanced to maintain osmotic balance.

Many terpenoids are involved in different metabolic activities of plant shoot. The plant nutrient status is related to the terpene metabolism [58]. The changes of terpenoids contribute to the self-protection and resistance of plants. In this study, the content of terpenoids in shoot at DSD 3 was significantly higher than that at DSD 7, and more terpenoids were involved in the DEMs in shoot than in those in root. It indicates that drought stress has a greater effect on terpene synthesis in shoot than in root, and the impact is greater in the early stage of drought stress under CP.

The metabolic profiling of wheat shoot in CP treatment showed that at the early stage of drought (DSD 0–3), the effect of free amino acids and proline on osmotic regulation was greater than that of soluble sugars (S1 Table). With the prolongation of drought stress, the photosynthesis, amino acid synthesis, proline metabolism were gradually suppressed by drought stress, while the sugar metabolism and organic acids were increased. At this time, the effect of soluble sugars on osmotic regulation was greater than that of proline (S1 Table). Besides, the ion content analysis showed that the contents of cations such as K^+^, Ca^2+^ and Na^+^ in shoot was significantly higher in CP treatment than in LP treatment at DSD 7. Previous studies have shown that the accumulation of organic acids in vacuoles may play a central role in regulating intracellular pH by neutralizing excess cations [59]. Therefore, the accumulation of various organic acids in plant cells is necessary for plants under stress, which is a key adaptive strategy to maintain intracellular ion homeostasis. This shows that different osmolytes play different roles in different drought stages. However, in this study, due to the reduced sensitivity to drought, the change of osmolytes in shoot was not obvious in LP treatment.

Xanthohumol is a flavonoid that inhibits the proliferation of cancer cells [60]. It has been widely used in medical research. However, little research has been done on it in plants. Since flavonoids can scavenge free radicals and have antioxidant effects, it is speculated that the effect of xanthohumol is similar to that of SOD. Our results showed that 0.1% xanthohumol addition promoted the shoot growth. This indicates that xanthohumol can promote the transportation of assimilates to the shoot under stress, and play an important role in maintaining plant shoot vitality and water status.

## Conclusion

This study systematically investigated the responses of wheat to drought stress and rehydration under two phosphorus levels from the aspects of root morphology, physiology and biochemistry, ion content, and metabolism. It was found that appropriate low phosphorus supply could enhance the drought tolerance of wheat. When subjected to drought stress, the root system of wheat under low phosphorus supply became denser and the response to oxidative stress became weaker. At the same time, the carbohydrate metabolism is greatly enhanced to regulate osmotic balance, and the accumulation of various organic acids is also improved to maintain intracellular ion homeostasis and enhance drought tolerance. These ultimately contribute to the fast recovery of wheat after rehydration.

## Supporting information

S1 File Metabolomic data in shoot and root of wheat. (XLSX)

S2 File Heat map of metabolites in root and shoot of wheat in CP and LP treatments. (PDF)

S1 Table Pathways enriched by differentially expressed metabolites in shoots of Xindong 20 in conventional phosphorus treatment at day 3 and 7 under simulated drought stress. (PDF)

S2 Table Pathways enriched by differentially expressed metabolites in roots of Xindong 20 in conventional phosphorus treatment at day 3 and 7 under simulated drought stress. (PDF)

S3 Table Pathway comparison with significant differences in the roots of Xindong 20 in low phosphorus treatment at day 3 and 7 under simulated drought stress. (PDF)

## Abbreviations

CP: conventional phosphorus
LP: low phosphorus
DSD 3: Day 3 under simulated drought stress
DSD 5: Day 5 under simulated drought stress
DSD 7: Day 7 under simulated drought stress
RD 3: Day 3 after rehydration
MDA: malondialdehyde
SOD: superoxide dismutase
POD: peroxidase
