# Supplementary material for: Ionomic and metabolic responses of wheat seedlings to PEG-6000-simulated drought stress under two phosphorus levels
Source: PLoS One. 2022 Sep 20;17(9):e0274915. doi: 10.1371/journal.pone.0274915 (PMC9488835; doi:10.1371/journal.pone.0274915)

Heat map of metabolites in the root of wheat in CP and LP treatments at DSD 3

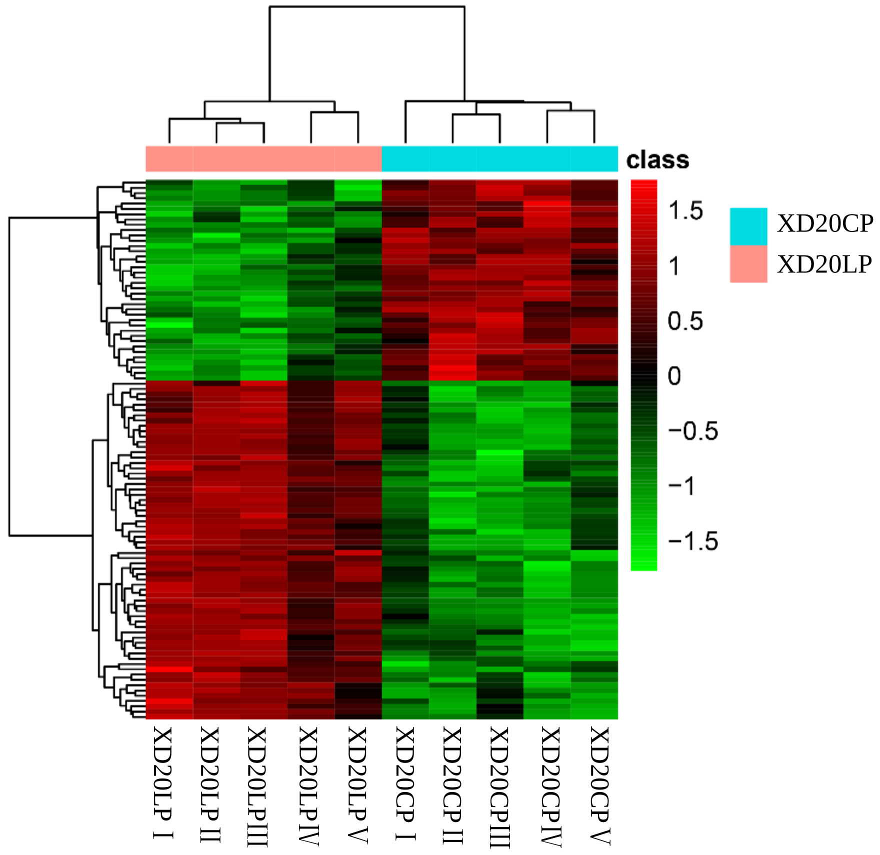

Heat map of metabolites in the root of wheat in CP and LP treatments at DSD 7

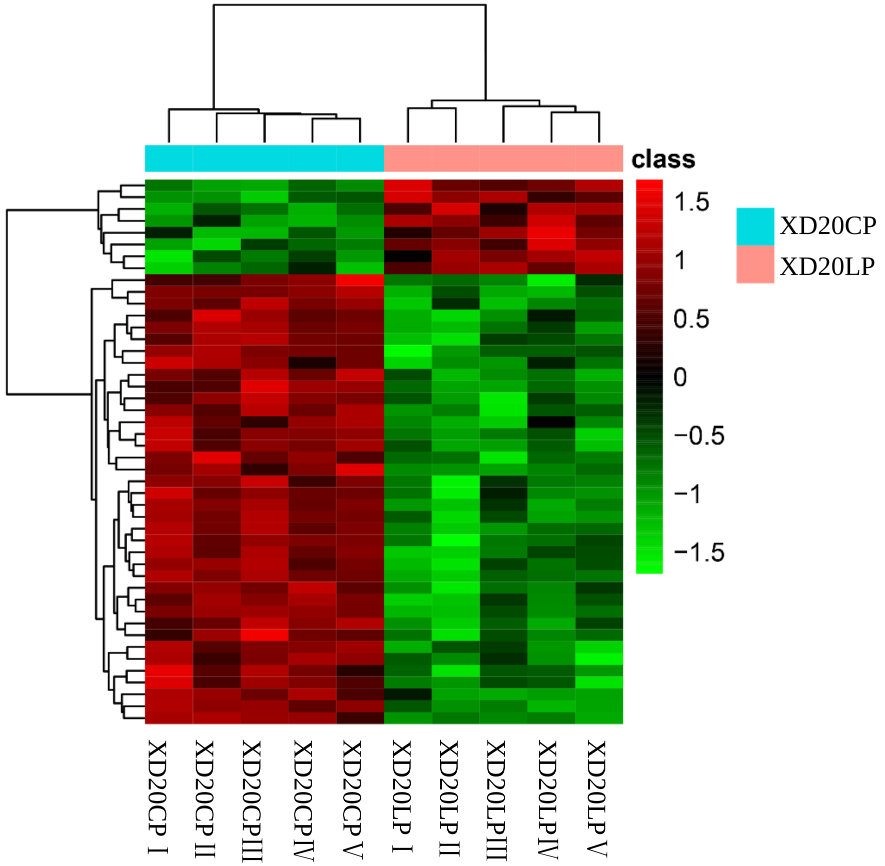

Heat map of metabolites in the shoot of wheat in CP and LP treatments at DSD 3

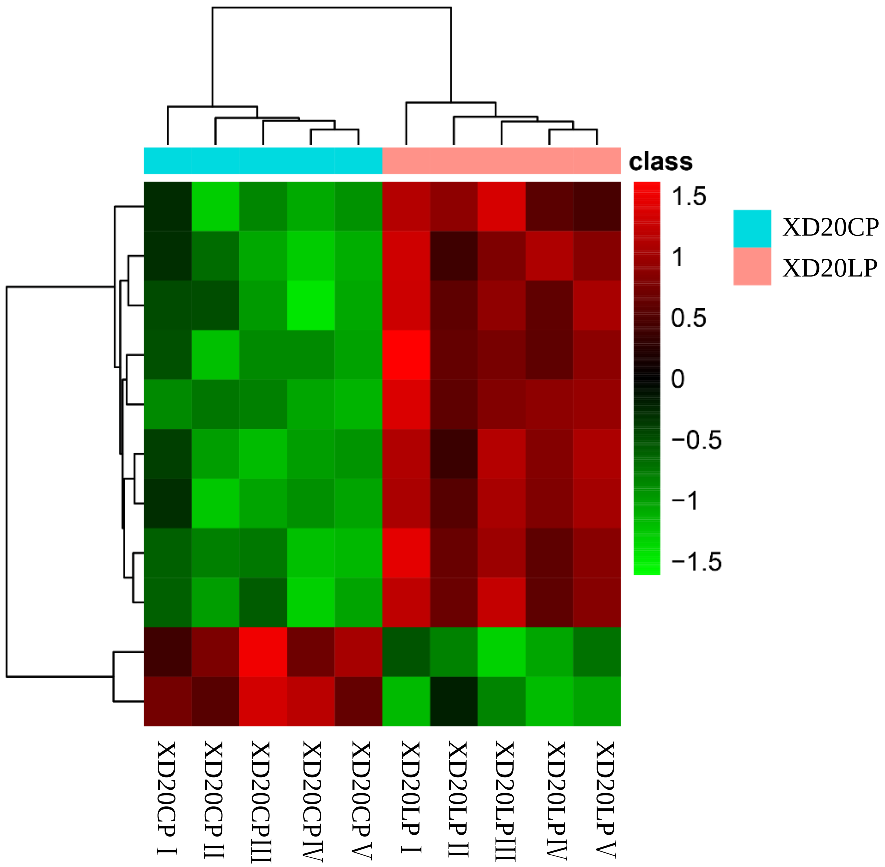

Supplement: S2 File — (PDF) [file pone.0274915.s002.pdf]
