# Supplementary material for: Ionomic and metabolic responses of wheat seedlings to PEG-6000-simulated drought stress under two phosphorus levels
Source: PLoS One. 2022 Sep 20;17(9):e0274915. doi: 10.1371/journal.pone.0274915 (PMC9488835; doi:10.1371/journal.pone.0274915)
Supplement: S1 Table — (PDF) [file pone.0274915.s003.pdf]

S1 Table Pathways enriched by differentially expressed metabolites in shoots of Xindong 20 in conventional phosphorus treatment at day 3 and 7 under simulated drought stress.

Pathway	Count	Pathway	Count	Pathway	Count
Purine metabolism	2	Tryptophan metabolism	3	Sphingolipid metabolism	2
Sulfur metabolism	1	alpha-Linolenic acid metabolism	16	Pantothenate and CoA biosynthesis	3
Biosynthesis of amino acids	6	Terpenoid backbone biosynthesis	3	Fatty acid metabolism	2
Starch and sucrose metabolism	7	Cyanoamino acid metabolism	1	Mannose type O-glycan biosynthesis	1
Monobactam biosynthesis	2	Sesquiterpenoid and triterpenoid biosynthesis	47	Flavone and flavonol biosynthesis	4
One carbon pool by folate	1	ABC transporters	3	Sulfur relay system	1
Flavonoid biosynthesis	1	Steroid biosynthesis	2	Ascorbate and aldarate metabolism	3
Nicotinate and nicotinamide metabolism	1	Biosynthesis of unsaturated fatty acids	6	Valine, leucine and isoleucine degradation	1
N-Glycan biosynthesis	4	Photosynthesis	1	Isoflavonoid biosynthesis	4
Ubiquinone and other terpenoid-quinone biosynthesis	9	Amino sugar and nucleotide sugar metabolism	8	Histidine metabolism	4
Glucosinolate biosynthesis	3	Brassinosteroid biosynthesis	5	Cutin, suberine and wax biosynthesis	2
Glutathione metabolism	2	Carotenoid biosynthesis	35	Glyoxylate and dicarboxylate metabolism	1
Phenylalanine metabolism	1	Linoleic acid metabolism	2	2-Oxocarboxylic acid metabolism	3
Glycerophospholipid metabolism	1	Tyrosine metabolism	2	Biosynthesis of secondary metabolites	126
Cysteine and methionine metabolism	4	Valine, leucine and isoleucine biosynthesis	1	Lysine degradation	1
Isoquinoline alkaloid biosynthesis	9	Betalain biosynthesis	2	Plant hormone signal transduction	2
Arginine and proline metabolism	2	Anthocyanin biosynthesis	12	Biotin metabolism	1
Phosphonate and phosphinate metabolism	1	Arachidonic acid metabolism	44	Aminoacyl-tRNA biosynthesis	1
Carbapenem biosynthesis	2	Porphyrin and chlorophyll metabolism	15	Galactose metabolism	1
Stilbenoid, diarylheptanoid and gingerol biosynthesis	4	Indole alkaloid biosynthesis	4	Pyrimidine metabolism	2
Fructose and mannose metabolism	1	Monoterpenoid biosynthesis	1	Diterpenoid biosynthesis	20
Taurine and hypotaurine metabolism	1	Metabolic pathways	126		
Zeatin biosynthesis	3	Tropane, piperidine and pyridine alkaloid biosynthesis	6		
