# Supplementary material for: Ionomic and metabolic responses of wheat seedlings to PEG-6000-simulated drought stress under two phosphorus levels
Source: PLoS One. 2022 Sep 20;17(9):e0274915. doi: 10.1371/journal.pone.0274915 (PMC9488835; doi:10.1371/journal.pone.0274915)
Supplement: S2 Table — (PDF) [file pone.0274915.s004.pdf]

S2 Table Pathways enriched by differentially expressed metabolites in roots of Xindong 20 in conventional phosphorus treatment at day 3 and 7 under simulated drought stress.

Pathway	Count	Pathway	Count	Pathway	Count
Biotin metabolism	1	Metabolic pathways	4	Biosynthesis of secondary metabolites	3
Cutin, suberine and wax biosynthesis	1	Indole alkaloid biosynthesis	1	ABC transporters	4
Starch and sucrose metabolism	4	Galactose metabolism	2		
