# Supplementary material for: Ionomic and metabolic responses of wheat seedlings to PEG-6000-simulated drought stress under two phosphorus levels
Source: PLoS One. 2022 Sep 20;17(9):e0274915. doi: 10.1371/journal.pone.0274915 (PMC9488835; doi:10.1371/journal.pone.0274915)
Supplement: S3 Table — (PDF) [file pone.0274915.s005.pdf]

S3 Table Pathway comparison with significant differences in the roots of Xindong 20 in low phosphorus treatment at day 3 and 7 under simulated drought stress.

Pathway	Count	Pathway	Count	Pathway	Count
Butanoate metabolism	4	Ascorbate and aldarate metabolism	4	Pyrimidine metabolism	2
Carbon metabolism	2	Isoquinoline alkaloid biosynthesis	2	Pentose phosphate pathway	1
Arginine biosynthesis	1	Amino sugar and nucleotide sugar metabolism	2	Phenylalanine metabolism	2
Nicotinate and nicotinamide metabolism	2	Tryptophan metabolism	1	Glyoxylate and dicarboxylate metabolism	1
Pyruvate metabolism	2	Tyrosine metabolism	3	Pentose and glucuronate interconversions	10
Isoflavonoid biosynthesis	1	Valine, leucine and isoleucine biosynthesis	2	Biosynthesis of secondary metabolites	10
Galactose metabolism	1	Purine metabolism	3	Limonene and pinene degradation	4
Oxidative phosphorylation	1	Glucosinolate biosynthesis	2	Arginine and proline metabolism	1
β-Linolenic acid metabolism	6	Folate biosynthesis	1	Glycerolipid metabolism	1
Metabolic pathways	23	Sulfur relay system	1	Alanine, aspartate and glutamate metabolism	1
ABC transporters	4	Citrate cycle (TCA cycle)	2	Ubiquinone and other terpenoid-quinone biosynthesis	2
Plant hormone signal transduction	1	Histidine metabolism	1	2-Oxocarboxylic acid metabolism	4
Carbon fixation in photosynthetic organisms	1	C5-Branched dibasic acid metabolism	6	Biosynthesis of amino acids	2
